# Carriage of *Streptococcus pneumoniae* in Aged Adults with Influenza-Like-Illness

**DOI:** 10.1371/journal.pone.0119875

**Published:** 2015-03-19

**Authors:** Cassandra L. Krone, Anne L. Wyllie, Josine van Beek, Nynke Y. Rots, Anna E. Oja, Mei Ling J. N. Chu, Jacob P. Bruin, Debby Bogaert, Elisabeth A. M. Sanders, Krzysztof Trzciński

**Affiliations:** 1 Paediatric Immunology and Infectious Diseases, Wilhelmina Children’s Hospital, University Medical Center Utrecht, Utrecht, The Netherlands; 2 Centre for Infectious Disease Control Netherlands, National Institute for Public Health and the Environment (RIVM), Bilthoven, The Netherlands; 3 Regional Laboratory of Public Health, Haarlem, The Netherlands; Faculdade de Medicina de Lisboa, PORTUGAL

## Abstract

Incidence of pneumococcal disease is disproportionally high in infants and elderly. Nasopharyngeal colonisation by *Streptococcus pneumoniae* is considered a prerequisite for disease but unlike in children, carriage in elderly is rarely detected. Here, we tested for *S*. *pneumoniae* in nasopharyngeal and saliva samples collected from community-dwelling elderly with influenza-like-illness (ILI). Trans-nasal nasopharyngeal, trans-oral nasopharyngeal and saliva samples (n = 270 per sample type) were collected during winter/spring 2011/2012 from 135 persons aged 60–89 at onset of ILI and 7–9 weeks later following recovery. After samples were tested for pneumococci by conventional culture, all plate growth was collected. DNA extracted from plate harvests was tested by quantitative-PCRs (qPCR) specific for *S*. *pneumoniae* and serotypes included in the 13-valent pneumococcal conjugated vaccine (PCV13). Pneumococci were cultured from 14 of 135 (10%) elderly with none of the sampled niches showing superiority in carriage detection. With 76/270 (28%) saliva, 31/270 (11%) trans-oral and 13/270 (5%) trans-nasal samples positive by qPCR, saliva was superior to nasopharyngeal swabs (p<0.001) in qPCR-based carriage detection. Overall, from all methods used in the study, 65 of 135 (48%) elderly carried pneumococci at least once and 26 (19%) at both study time points. The difference between carriage prevalence at ILI (n = 49 or 36%) versus recovery (n = 42 or 31%) was not significant (p = 0.38). At least 23 of 91 (25%) carriage events in 19 of 65 (29%) carriers were associated with PCV13-serotypes. We detected a large reservoir of pneumococci in saliva of elderly, with PCV13-serotype distribution closely resembling the contemporary carriage of serotypes reported in the Netherlands for PCV-vaccinated infants.

## Introduction

*Streptococcus pneumoniae* is a frequent but transient commensal of the human upper respiratory tract (URT) that can progress to respiratory and invasive pneumococcal disease (IPD) [1]. Disease disproportionally affects the very young and the elderly [2]. Pneumococcal conjugate vaccines (PCV), targeting up to 13 (PCV13) from over 90 [3] known pneumococcal serotypes are now widely available, with vaccination recommended in the first two years of life and in those deemed to be at risk of IPD [4]. Carriage of *S*. *pneumoniae* in the URT is considered a prerequisite for pneumococcal disease, therefore surveillance studies provide insight into the prevalence of carriage and serotypes circulating in the population in order to assess direct and herd effects of vaccine implementation [5]. While vaccination of elderly persons has been considered [4], it has been assumed that immunisation of infants alone may have already resulted in herd immunity, protecting adults against disease caused by vaccine serotypes (VTs) [6]. However, studies investigating the herd effects of PCVs on serotype carriage in adults are limited and even more rare when elderly are considered [7–9].

Currently, the gold standard for pneumococcal carriage detection in children is the isolation of live *S*. *pneumoniae* following conventional culture of deep trans-nasal nasopharyngeal swabs [10]. In adults, the addition of a trans-orally obtained nasopharyngeal or oropharyngeal swab significantly increases carriage detection [10,11]. Furthermore, culture-independent diagnostic methods have largely improved the sensitivity of *S*. *pneumoniae* carriage detection in nasopharyngeal samples from both children [12–14] and adults [11,15]. Interestingly, in the early 1900s the consensus was that the pneumococcus was carried in the saliva of 45–60% of all healthy adults, including elderly [16]. *S*. *pneumoniae* was detected in these historical studies with sensitive animal inoculation methods [16–19]. During the time between the dawn of the antibiotic era in the mid-20^th^-century and the introduction of PCVs, carriage studies in adults and elderly were rarely performed [7]. Moreover, the method of detecting pneumococcus in the URT moved from saliva to nasopharyngeal swabs, due in part to the highly polymicrobial nature of saliva, making isolation of *S*. *pneumoniae* from culture very difficult [20,21]. In the scarce recent studies among elderly, the use of swabs usually resulted in carriage being detected in less than 5% of individuals [8,9,22–25]. Low rates of nasopharyngeal pneumococcal carriage argue against significant benefits of epidemiological surveillances on colonisation in this age group [7,8].

We hypothesised that testing nasopharyngeal swabs by conventional culture methods alone underestimates the prevalence of *S*. *pneumoniae* carriage in aged adults. Recent advances in molecular detection methods prompted us to revisit saliva as a diagnostic specimen in epidemiological studies in an attempt to improve detection of pneumococcal carriage in this age group. We compared culture and molecular-based methods for the detection of *S*. *pneumoniae* in the elderly on trans-nasal nasopharyngeal, trans-oral nasopharyngeal and saliva samples and investigated the carriage of PCV13-serotypes within this population. Viral respiratory tract infections are associated with an increased risk of pneumococcal pneumonia and IPD [26–28], therefore we hypothesised that rates and density of pneumococcal carriage are higher in elderly with symptoms of influenza-like-illness (ILI) compared after their recovery from ILI, 7–9 weeks later.

We provide evidence for the superiority of saliva sampling for pneumococcal carriage detection in aged adults and conclude that current rates of *S*. *pneumoniae* carriage in the elderly might be largely underestimated. Furthermore, we provide evidence of substantial carriage of PCV13-serotypes and longitudinal carriage of the same serotype in elderly. However, we found no evidence of higher pneumococcal carriage rates nor density at ILI compared to after recovery.

## Materials and Methods

### Influenza-like-illness in elderly study

To assess the incidence and cause of ILI among elderly in the Netherlands, an open cohort observational study was performed among community-dwelling adults aged ≥60 years during autumn/winter 2011/2012. Of 21,000 elderly contacted by open invitation by post, 2,120 consented to participate. Of these, 146 participants were eligible for first sampling, reporting with ILI symptoms defined by Pel [29] as fever >37.8ºC (rectal) in combination with at least one of the following: rhinitis, cough, sore throat, headache, myalgia, chest pain. Written informed consent was obtained from all participants and the study was conducted in compliance with Good Clinical Practice and the Declaration of Helsinki of the World Medical Association. The study was approved by an acknowledged Dutch medical ethics committee METC Noord Holland (NTR 3386). Demographic information was collected from study participants at the first visit.

### Sample collection

Individuals were sampled twice: first at the onset of ILI and then 7–9 weeks later, after recovery. Trained personnel collected all samples during home visits. Deep trans-nasal nasopharyngeal samples were obtained using flexible swabs according to the World Health Organisation standard procedure [10]. Trans-oral nasopharyngeal samples were collected with rigid swabs under direct observation of the posterior pharynx [11]. Swabs were placed individually in 1 ml Amies medium (Copan, Brescia, Italy) at room temperature. Saliva was collected with the Oracol Saliva Collection System (Malvern Medical Developments Limited, Worcester, UK), immediately transferred to tubes pre-filled with 100 μl sterile 50% glycerol solution in water and placed on dry ice for transport. With approximately 400 μl of saliva collected per sample the final glycerol concentration was around 10%. All samples were transferred within 8 hours to the Regional Laboratory of Public Health in Haarlem.

### Culture of samples

On arrival, 10 μl of the trans-oral sample was cultured on trypticase soy agar supplemented with 7% defibrinated sheep blood and gentamicin 5 mg/l (SB7-Gent, Oxoid, Badhoevedorp, Netherlands) and processed for *S*. *pneumoniae* detection by the standard culture diagnostic approach [30]. All bacterial growth was harvested from SB7-Gent plates into 10% glycerol in BHI (Oxoid) and stored frozen as previously described [11]. Trans-nasal samples were supplemented with 10% glycerol and stored frozen. Later, trans-nasal and saliva samples were thawed and 10 μl of trans-nasal samples and 100 μl of saliva were cultured on SB7-Gent plates and processed as for the trans-oral samples. SB7-Gent plate harvests are hereafter considered as culture-enriched samples [11]. Culture-enriched samples identified as positive for pneumococcus by molecular methods were re-cultured in an attempt to recover live pneumococci as previously described [11,21]. All *S*. *pneumoniae* strains cultured were stored for serotype determination.

### Isolation of DNA

DNA was extracted from 100 μl of raw saliva samples and from 200 μl of all culture-enriched samples as previously described [21].

### Molecular detection of *S. pneumoniae*

*S. pneumoniae*-specific DNA was detected by quantitative-PCR (qPCR) targeting pneumococcal genes *lytA* [31] and *piaA* [11]. Samples were considered positive for *S*. *pneumoniae* when C_*T*_ values for both genes were <40 [21].

### Sample serotype determination using molecular methods

Sample serotype composition was determined for all DNA templates using a panel of primers and probes, targeting PCV13-serotypes 1, 3, 4, 5, 14, 19A, 23F [13] and 19F [32], and serogroups 6A/B, 7A/F, 9A/V [13] and 18A/B/C/F [32]. Samples were considered positive for the serotype or serogroup when the serotype/serogroup-specific signal was <40 C_*T*_ [33]. Only type-specific qPCR assays that did not generate any positive results for samples negative for *S*. *pneumoniae* were considered reliable for use in the study [21].

### Quellung reaction

*S*. *pneumoniae* strains were serotyped at the Regional Laboratory of Public Health in Haarlem using the Quellung method and serotype-specific sera (Staten Serum Institut, Copenhagen, Denmark).

### Statistical methods

Statistical analyses were conducted using GraphPad Prism v5.0 (GraphPad Software, San Diego, CA, USA). Statistical differences were detected using two-way Fisher’s exact tests for 2×2, or Chi-square for 3×2 contingency tables. The relationship between quantitative data for *lytA* and *piaA* detection was evaluated with non-parametric Spearman correlation. Statistical significance was defined as *P*<0.05.

## Results

**Fig 1:**
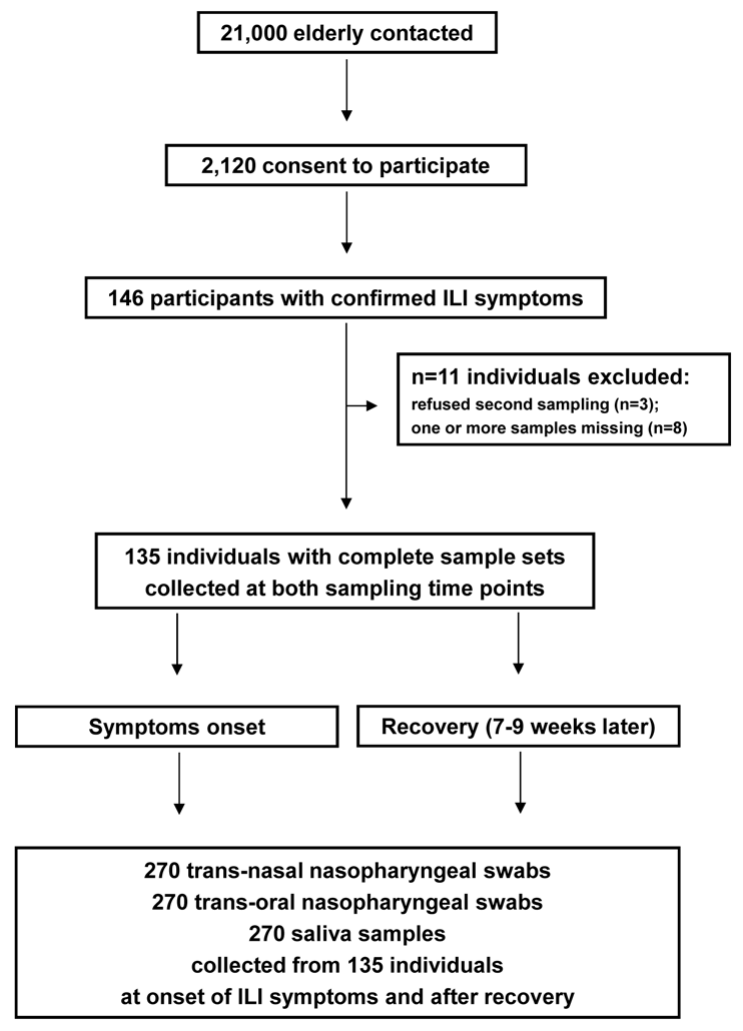
Enrolment and samples obtained in this study. In total 810 samples were analysed.

Of 146 elderly who reported with ILI, 135 (92%) persons (59% female, mean age 69 years) and 270 samples per niche were analysed in the study (Fig. 1).

### Isolation of *S. pneumoniae* from cultures

**Fig 2:**
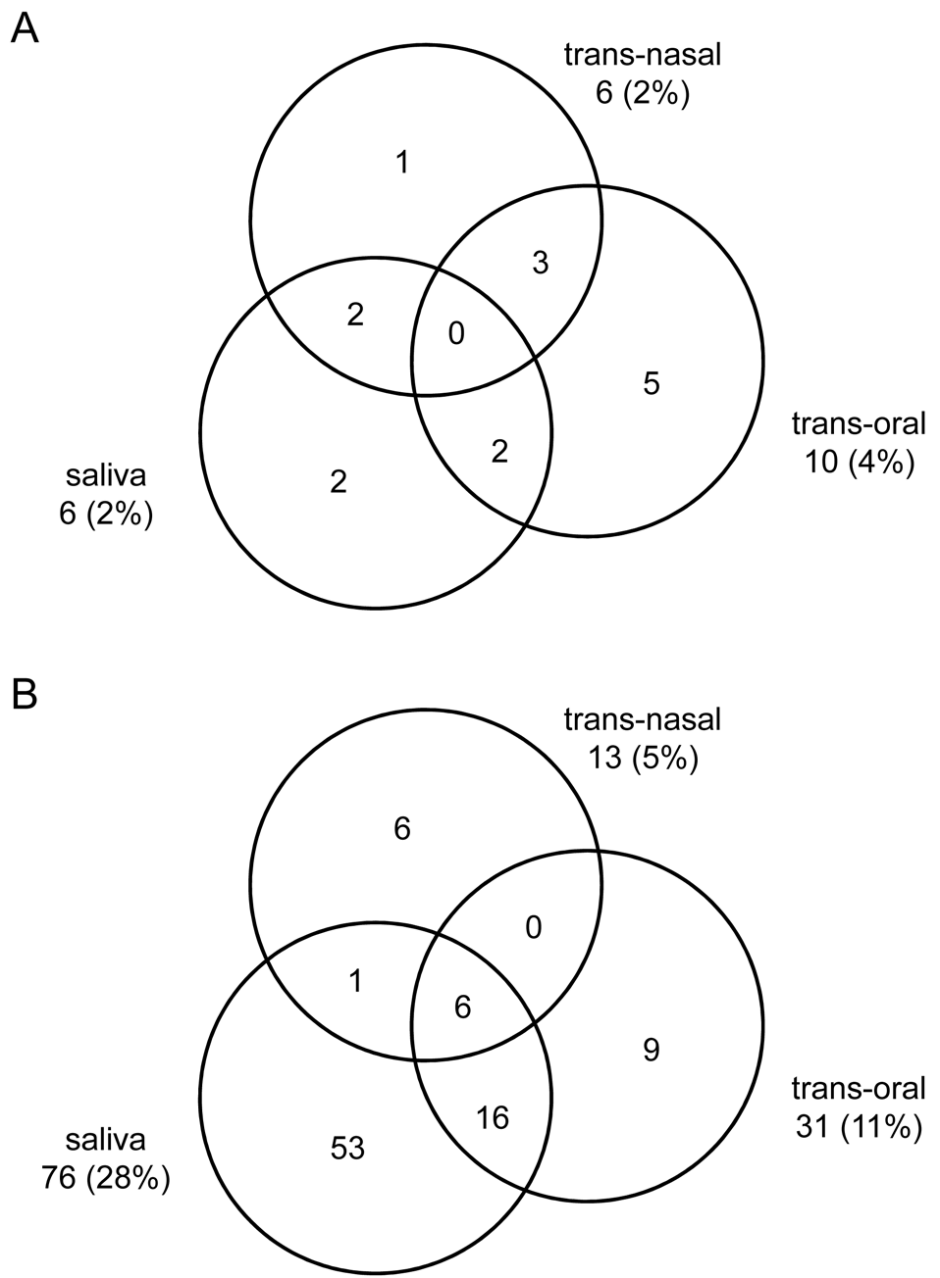
Detection of *Streptococcus pneumoniae* in all samples analysed in the study. Figure shows the distribution of samples classified in the study as positive for *S*. *pneumoniae* based on (A) isolation of live *S*. *pneumoniae* from cultured samples or (B) detection of *S*. *pneumoniae* by qPCR in DNA extracted from culture-enriched samples of trans-nasal swabs (n = 270), culture-enriched trans-oral swabs (n = 270) and culture-enriched saliva samples (n = 270) tested. Each circle represents sample type as labelled. Numbers next to sample type depict number (% of 270) of positive samples. Overlapping areas depict matching positive samples (detection of *S*. *pneumoniae* simultaneously in more than one of three samples collected per sampling event).

Live *S. pneumoniae* were isolated from only two (1%) of 270 trans-oral samples by conventional culture. After re-culturing of culture-enriched samples determined to be positive for *S*. *pneumoniae* by the molecular method, live pneumococci were isolated from six (2%) of 270 trans-nasal, six (2%) of 270 saliva samples and the number of trans-oral samples culture-positive for *S*. *pneumoniae* increased to ten (4%) of 270 (Fig. 2A). Differences between niches were not significant (Fisher’s exact, p = 0.45). In total, all cultures combined identified 14 (10%) of 135 elderly with pneumococcal carriage; eight only during ILI, five only post-recovery and one at both sampling events. The difference in the number of carriers culture-positive for *S*. *pneumoniae* between ILI (n = 9) and after recovery (n = 6) was not significant (p = 0.45).

### Molecular detection of *S. pneumoniae*

Thirteen (5%) of 270 culture-enriched trans-nasal samples, 31 (11%) of 270 culture-enriched trans-oral samples and 76 (28%) of 270 culture-enriched saliva samples were qPCR-positive for *S. pneumoniae* (Chi-square, p<0.0001) (Fig. 2B). Compared to DNA extracted from uncultured saliva samples, culture-enrichment significantly increased the number of samples classified as positive for *S*. *pneumoniae* by the molecular method (S1 Fig.). All samples culture-positive for pneumococcus were also identified as positive by qPCR.

### Optimal niche for detection of pneumococcal carriage in elderly

**Fig 3:**
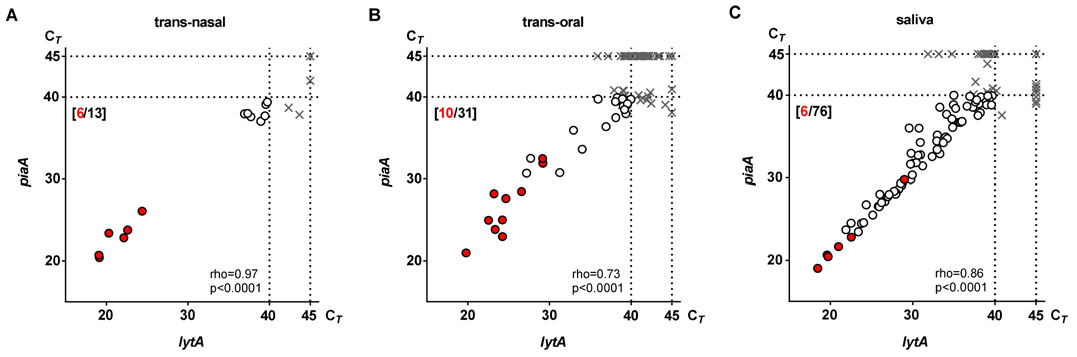
qPCR-based detection of *Streptococcus pneumoniae* versus isolation of live pneumococci from trans-nasal (n = 270), trans-oral (n = 270) and saliva (n = 270) samples analysed in the study. Figure depicts results of qPCR-based detection of *S*. *pneumoniae-*specific genes *lytA* and *piaA* and results of live *Streptococcus pneumoniae* isolation from culture-enriched trans-nasal samples (A), culture-enriched trans-oral samples (B) and culture-enriched saliva samples (C) from 135 elderly. Each symbol (dot or cross) represents an individual sample. Position of the symbol corresponds to C_*T*_ values for *lytA*- and *piaA*-specific signals as marked on corresponding axes. Red dots represent samples from which live pneumococci were isolated by conventional culture at primary diagnostic step or from re-culture of samples qPCR-positive for *S*. *pneumoniae*. Open dots represent samples classified with qPCR as positive yet culture-negative for *S*. *pneumoniae*. Crosses represent samples classified as negative for *S*. *pneumoniae* in the study. Dotted lines mark the threshold of sample positivity based on presence of signals for both *lytA* and *piaA* C_*T*_ <40 and the continuous lines represent total number of 45 cycles in each qPCR reaction. Number depicts number of samples identified as positive for *S*. *pneumoniae* by culture (in red) and by molecular method (in black). Spearman’s rank correlation coefficient (rho) and the associated *P* value (p) are shown.

Altogether, carriage of *S*. *pneumoniae* was detected by culture in 15 (6%) and by molecular method in 91 (34%) of the 270 triads of samples collected from the 135 elderly, at ILI and recovery combined (Fig. 3). The number of trans-nasal samples qPCR-positive for *S*. *pneumoniae* (13 of 270) was not significantly higher compared to the number positive by culture (six of 270; p = 0.45). For both trans-oral (31 vs. ten of 270; p<0.01) and saliva (76 vs. six of 270; p<0.0001) the number of samples identified as positive for *S*. *pneumoniae* by qPCR was significantly higher when compared to culture. Overall, qPCR-based detection of *S*. *pneumoniae* in culture-enriched saliva samples was the most sensitive method of carriage detection in this study (Table 1). Limiting the detection of pneumococci to the molecular analyses of culture-enriched saliva alone without testing nasopharyngeal samples would decrease the detected carriage rate from 34% to 28% (91 vs. 76 of 270, p = 0.16), whereas not including saliva samples in the study would lower detection from 34% to 14% (91 vs. 38 of 270, p<0.001).

**Table 1:** Sensitivity of methods used to detect *Streptococcus pneumoniae* carriage in this study.

Method of *S*. *pneumoniae* detection	Number (%)^a^ of detected carriage events	Sensitivity of method ^b^
Conventional culture		
trans-nasal swab	6 (2)	0.07
trans-oral swab	10 (4)	0.11
saliva	6 (2)	0.07
Molecular detection in culture-enriched sample		
trans-nasal swab	13 (5)	0.14
trans-oral swab	31 (11)	0.34
either trans-nasal or trans-oral swab	38 (14)	0.42
culture-enriched saliva	76 (28)	0.84

^a^Fraction of 270 samples of particular type processed in the study.

^b^Fraction of 91 *S*. *pneumoniae* carriage events identified by any method in the study.

### Point and period prevalence of pneumococcal carriage in elderly

We collected samples from individuals at two time points, at the onset of ILI symptoms and after recovery. Based on the combined results of pneumococcal carriage detection by any method in the study, the carriage prevalence for ILI onset was 49 (36%) of 135 and for post-recovery was 42 (31%) of 135. These differences were not statistically significant (p = 0.37). In total, 65 individuals were positive for *S*. *pneumoniae* in at least one sample collected during either sampling event, resulting in a period prevalence of 48% in this population of elderly (Table 2). Twenty-six (19%) elderly were identified as pneumococci-positive at both time points. There was no difference in density of *S*. *pneumoniae* detected with qPCR in DNA extracted from raw saliva samples collected at ILI versus recovery (Mann-Whitney, p = 0.36; S2 Fig.).

**Table 2:** Patient characteristics related to *Streptococcus pneumoniae* carriage detected in the study.

Patient groups		Number of persons	Number (%) of persons positive for *S*. *pneumoniae* at least once in the study	Number (%) of persons positive for *S*.* pneumoniae* at both study time points	Number of sampling events	Number (%) of sampling events positive for *S*. *pneumoniae*
Total		135	65 (48)	26 (19)	270	91 (34)
Sex	Female	80	36 (45)	12 (15)	160	48 (30)
	Male	55	29 (53)	14 (25)	110	43 (39)
Age (years)	81–90	12	5 (42)	2 (17)	24	7 (29)
	71–80	37	12 (32)	5 (14)	74	17 (23)
	60–70	86	48 (56)	19 (22)	172	67 (39)

### Carriage of *S. pneumoniae* by age category and by sex

All positive samples were stratified by age and sex. Neither factor significantly affected pneumococcal detection (Table 2).

### Serotype carriage in the elderly

**Fig 4:**
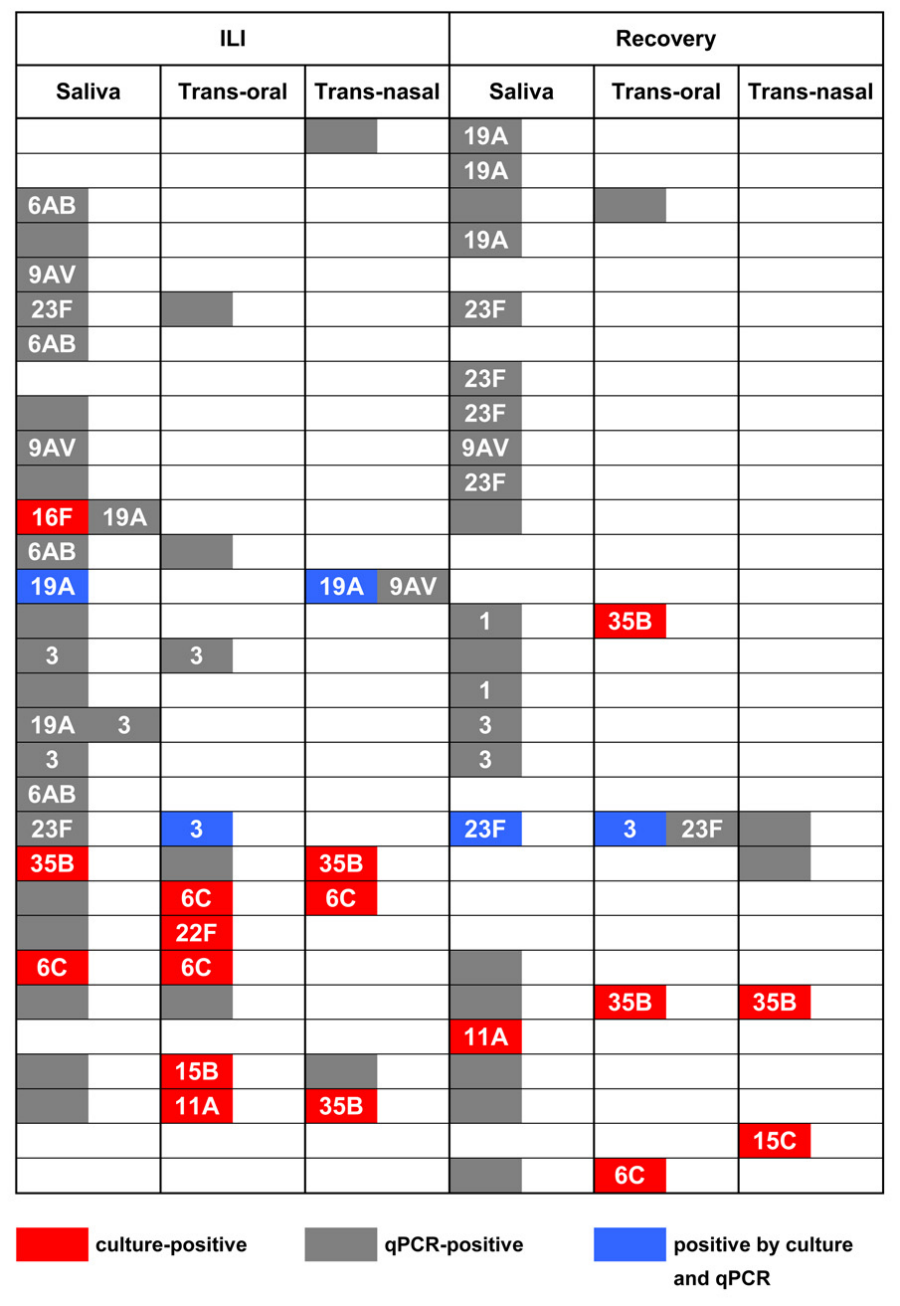
*Stretococcus pneumoniae* serotypes of pneumococcal strains detected in the study. Each row represents one of 31 individual study subjects either culture-positive for *S*. *pneumoniae* in any of the six samples collected in the study, or positive for carriage of PCV13-serotypes detected by qPCR (results for remaining 34 carriers of *S*. *pneumoniae* not shown). Serotype of cultured strain or serotype-specific signal detected depicted. Empty grey squares depict samples positive for *S*. *pneumoniae* by qPCR but negative for any of the serotypes tested for in the study.

We isolated 22 pneumococcal strains from 22 cultures positive for *S*. *pneumoniae* from 14 individuals in 15 sampling events (Fig. 3). In five (33%) of 15 sampling events strains of the same serotype were cultured from two different sample types. Strains of different serotypes were cultured in two (13%) of 15 sampling events. Based solely on the serotype of pneumococcal isolates determined using the Quellung method, an overall number of 17 *S*. *pneumoniae* strains were cultured in the study (Fig. 4).

DNA templates from all culture-enriched samples were tested in qPCR for serotypes targeted by PCV13. Following previously described criteria [21], results for qPCR assays specific for serotype 4 and 5 were excluded from analysis due to the presence of false-positive results. Of the remaining PCV13-serotypes, no sample was positive for serotypes 7F, 14, 18C, or 19F, however the presence of serotypes 1, 3, 6A/B, 9A/V, 19A, or 23F was detected in 26 (29%) of 91 carriage events in 21 (32%) of 65 carriers (Fig. 4). This includes 13 (27%) of 49 carriage events at ILI onset and 13 (31%) of 42 carriage events after recovery. Since the molecular method does not allow for distinction between VT 9V and NVT 9A the samples positive for 9A/V-specific signal could contribute to an overestimation of PCV13-serotype presence. On the other hand exclusion of all results for serotype 4 and 5 could lead to an underestimation of these two VTs in the study. The molecular method also does not allow for the distinction between 6A (targeted only by PCV13) and VT 6B. Taking these limitations into account, we interpreted the results from the molecular method for sample serotyping as evidence of at least, nine (10%) of 91 carriage events in seven (11%) of 65 carriers being associated with serotypes targeted by the 10-valent vaccine (PCV10) and at least seven (8%) carriage events in five (8%) carriers with serotypes targeted by 7-valent vaccine (PCV7).

## Discussion

The major finding of this study is the high rate of pneumococcal carriage in aged adults. We found that sampling saliva of elderly significantly increased the detection of *S*. *pneumoniae* compared to trans-nasal and trans-oral sampling when carriage was detected using molecular methods. Moreover, the period prevalence was 48%, with 19% of the elderly being positive at both time points. Even in the time of herd immunity in the fifth year after implementation of PCV7 and one year after PCV7 replacement with PCV10 in the national immunisation programme (NIP) for children, carriage of PCV13-serotype strains was still present in more than a quarter of *S*. *pneumoniae* carriers among these community-dwelling elderly.

Our data on the high rate of pneumococcal presence in saliva of aged adults are in agreement with early epidemiological studies from the pre-antibiotic era reporting approximately half of all adults asymptomatically carrying *S*. *pneumoniae* in saliva [20,16]. The low sensitivity of nasopharyngeal swabs in elderly may be due to the lower density of nasopharyngeal carriage in adulthood as compared to children in which conventional cultures of the nasopharynx identify carriage rates of 40–90% [7]. Alternatively, changes in the anatomy of the URT in adults and elderly may cause difficulty in accessing the nasopharyngeal niche. With the presently observed high rates of pneumococcal carriage in aged adults in community settings it seems plausible to link a disappearance of the *S*. *pneumoniae* reservoir in elderly carriage in the past half-century to changes in diagnostic procedures rather than to the improvement of living conditions and public health, especially since pneumococcal pneumonia remains a major disease burden in elderly [2,20].

We demonstrated that conventional culture detects fewer carriers of *S*. *pneumoniae* than molecular-based methods when applied to any niche sampled by us. This finding of the low sensitivity of conventional culture is in agreement with surveillance studies performed in the 1930s where culture of swabs yielded the lower rates of detection (sensitivity of 47%) when compared to the inoculation of mice with the swab transport medium (sensitivity of 93%) [17]. We also observed the advantage of *S*. *pneumoniae* detection by qPCR in DNA extracted from culture-enriched compared to unprocessed saliva (S1 Fig.). This is in concordance with other studies in middle-aged adults [11] and in children [12,21] reporting an increase in pneumococcal carriage detection in samples from the upper airways tested with molecular methods after culture-enrichment. Our results suggest that saliva in combination with molecular diagnostic methods could be considered as the sole specimen for pneumococcal carriage detection in the elderly.

We did not observe significant differences between rates or density of pneumococcal carriage at ILI onset and post-recovery with 19% of elderly positive for *S*. *pneumoniae* at both time points. In animal models, viral respiratory infection increased both duration of pneumococcal colonisation and density of carriage in the acute phase of infection [34,35], with the peak in density lagging 3 to 7 days behind infection onset [35,36]. Thus, sampling at ILI onset possibly preceded shifts in carriage density. In humans, an elevated pneumococcal carriage was reported to be associated with respiratory virus co-infection in patients hospitalised due to respiratory tract infection [37,38]. With no hospitalisations in our study, it is also possible that ILI symptoms were too mild to affect carriage density. Interestingly, Webster and Hughes [39] reported 20% of asymptomatic adults to be oropharyngeal carriers of the same *S*. *pneumoniae* serotype for periods of three to 36 months. This suggests that the carriage rate observed in our study is independent from ILI. Follow-up studies with more sampling moments in asymptomatic community-dwelling elderly may increase our knowledge regarding carriage duration and period prevalence in aged adults.

In the current study, frequencies of PCV7, PCV10 and PCV13-serotypes detected among elderly carriers closely resemble the frequencies of corresponding pneumococcal serotypes reported in vaccinated infants in the surveillance study conducted in 2010–2011, 4.5 years after PCV7 implementation, but before PCV10 introduction in the Dutch NIP [40]. In the absence of data on serotype carriage in elderly prior to this study, it is difficult to determine whether these similarities represent herd effects of infant immunisation on VT carriage in aged adults in the Netherlands, or if VT carriage in elderly was substantially lower compared to infants already, prior to PCV7 introduction. Interestingly, the serotypes of the NVT strains cultured in the study mirror NVTs observed at the corresponding period of time in carriage in infants [40] and schoolchildren [21]. More studies are needed to further assess the herd effects of infant vaccination and to determine if any of the serotypes replacing VTs in the general population could emerge from the reservoir in the elderly.

A possible limitation of our study is that live *S*. *pneumoniae* could not be isolated from the vast majority of samples that were qPCR-positive. We attribute this to the highly polymicrobial nature of trans-oral and saliva samples, which show abundant growth on culture plates making pneumococci hardly detectable [11,21,41]. The discordance between culture- and molecular-based detection of *S*. *pneumoniae* was particularly striking for saliva where over ten times more samples were identified as qPCR-positive for the presence of pneumococci (n = 76) compared to culture-confirmed results (n = 6). There have been no reports however, of non-pneumococcal strains testing positive for *lytA* by the qPCR assay employed in this study, despite it being widely used [17,21,23,25]. Furthermore, by targeting another pneumococcal gene we increased the specificity of *S*. *pneumoniae* detection, ensuring that any discordance between culture and molecular methods is not due to false-positive signal in a single molecular test [11,21]. Moreover, all culture-positive carriage events were also confirmed by the molecular method.

Since we and others have reported on the poor specificity of certain molecular assays targeting serotype-specific capsular genes in *S*. *pneumoniae* [21,41,42], we followed rigorous criteria when interpreting serotype detection using qPCR in polymicrobial samples in this study. By testing all samples regardless of positivity for pneumococci we were able to identify assays generating false-positive results and excluded these from analysis. Here we only report on results from serotype-specific molecular assays identified as 100% specific. Although we did not culture *S*. *pneumoniae* from the majority of samples classified as positive by molecular methods, we believe that these results accurately reflect pneumococcal carriage in this study population.

In conclusion, we found a high prevalence of pneumococcal carriage in elderly when molecular-based methods were used. Furthermore, saliva sampling significantly increased detection of *S*. *pneumoniae* in the elderly. We observed longitudinal carriage of the same serotype and simultaneous carriage of multiple serotypes in our study population, features described for pneumococcal carriage in young children. As collection of saliva is easy and minimally invasive, future carriage studies in the elderly could consider using these methods.

## Supporting Information

S1 Fig Impact of culture-enrichment on *Streptococcus pneumoniae* gene *lytA* detection with qPCR in saliva samples (n = 270) from elderly. Each dot or cross represents an individual sample. The position of symbols corresponds to C_*T*_ values for l*ytA*-specific signals in DNA extracted from raw and culture-enriched sample of saliva as marked on corresponding axes. Dots represent 76 saliva samples classified as positive and crosses represent 194 saliva samples classified as negative for *S*. *pneumoniae* in the study. Open dots represent 32 saliva samples classified as positive for *S*. *pneumoniae* in both raw and culture-enriched samples. Green dots represent 44 samples classified as positive only after culture-enrichment. Dotted lines mark the threshold assigned to discriminate between positive (C_*T*_ <40) and negative samples, and the total number of 45 cycles in the qPCR reaction. There was a significantly higher number of saliva samples classified as positive for *S*. *pneumoniae* after culture-enrichment compared to raw saliva samples (76 or 29% versus 31 or 11%; Fisher’s exact, p<0.001). Culture-enrichment increased the signal strength of the genes targeted by qPCR, with an average overall increase of 2.10 C_*T*_ for the *lytA* gene (maximum observed increase of 23.11 C_*T*_) and of overall increase of 1.90 C_*T*_ for the *piaA* (maximum increase of 21.30 C_*T*_). In the subset of 76 saliva samples identified as positive either in raw or culture-enriched sample an average increase for *lytA* was 6.92 C_*T*_ and for *piaA* was 6.64 C_*T*_.
(PDF)

S2 Fig Absolute abundance of *Streptococcus pneumoniae* in saliva samples collected at ILI onset and after recovery from ILI. Each dot represents an individual sample. The position of symbols corresponds to C_*T*_ values for *lytA*-specific signals in DNA extracted from raw sample of saliva at ILI onset or after recovery, as marked on X-axis. Horizontal lines represent mean C_*T*_ value. There was no significant difference between quantities of *lytA* detected in samples collected at ILI (n = 135) versus the post-recovery sampling time point (n = 135; Mann-Whitney, p = 0.36), neither for the subset of samples considered in the study as positive for *S*. *pneumoniae* by qPCR at ILI (n = 42) versus post-recovery (n = 34; p = 0.26).
(PDF)

## References

[1] Simell B, Auranen K, Käyhty H, Goldblatt D, Dagan R, O’Brien KL. (2012) The fundamental link between pneumococcal carriage and disease. Expert Rev Vaccines 11: 841–55. 22913260 10.1586/erv.12.53

[2] Janssens J, Krause K. (2004) Pneumonia in the very old. Lancet Infect Dis 4: 112–24. 14871636 10.1016/S1473-3099(04)00931-4

[3] Park IH, Geno KA, Yu J, Oliver MB, Kim K-H, Nahm MH. (2015) Genetic, biochemical, and serological characterization of a new pneumococcal serotype, 6H, and generation of a pneumococcal strain producing three different capsular repeat units. Clin Vaccine Immunol (Epub ahead of print). 10.1128/CVI.00647-14 PMC4340893 25589550

[4] Aliberti S, Mantero M, Mirsaeidi M, Blasi F. (2014) The role of vaccination in preventing pneumococcal disease in adults. Clin Microbiol Infect 20 Suppl.5: 52–8. 10.1111/1469-0691.12518 24410778 PMC4473770

[5] Weinberger DM, Malley R, Lipsitch M. (2011) Serotype replacement in disease after pneumococcal vaccination. Lancet 378: 1962–73. 10.1016/S0140-6736(10)62225-8 21492929 PMC3256741

[6] Van Hoek AJ, Sheppard CL, Andrews NJ, Waight PA, Slack MPE, Harrison TG, et al (2014) Pneumococcal carriage in children and adults two years after introduction of the thirteen valent pneumococcal conjugate vaccine in England. Vaccine 20: 1–7. 10.1016/j.vaccine.2014.03.017 24657717

[7] Le Polain de Waroux O, Flasche S, Prieto-Merino D, Edmunds WJ. (2014) Age-dependent prevalence of nasopharyngeal carriage of *Streptococcus pneumoniae* before conjugate vaccine introduction: A prediction model based on a meta-analysis. PLoS One 9: e86136 10.1371/journal.pone.0086136 24465920 PMC3900487

[8] Almeida ST, Nunes S, Santos Paulo AC, Valadares I, Martins S, Breia F, et al (2014) Low prevalence of pneumococcal carriage and high serotype and genotype diversity among adults over 60 years of age living in Portugal. PLoS One 9: e90974. 24604030 10.1371/journal.pone.0090974 PMC3946249

[9] Hamaluba M, Kandasamy R, Ndimah S, Morton R, Caccamo M, Robinson H, et al (2015) A cross-sectional observational study of pneumococcal carriage in children, their parents, and older adults following the introduction of the 7-valent pneumococcal conjugate vaccine. Medicine 94: e335 10.1097/MD.0000000000000335 25569650 PMC4602851

[10] Satzke C, Turner P, Virolainen-Julkunen A, Adrian PV, Antonio M, Hare KM, et al (2013) Standard method for detecting upper respiratory carriage of *Streptococcus pneumoniae*: Updated recommendations from the World Health Organization Pneumococcal Carriage Working Group. Vaccine 32: 165–79. 10.1016/j.vaccine.2013.08.062 24331112

[11] Trzciński K, Bogaert D, Wyllie A, Chu MLJN, van der Ende A, Bruin JP, et al (2013) Superiority of trans-oral over trans-nasal sampling in detecting *Streptococcus pneumoniae* colonization in adults. PLoS One 8: e60520 10.1371/journal.pone.0060520 23555985 PMC3610877

[12] Carvalho MD, Pimenta FC, Jackson D, Roundtree A, Ahmad Y, Millar EV, et al (2010) Revisiting pneumococcal carriage by use of broth enrichment and PCR techniques for enhanced detection of carriage and serotypes. J Clin Microbiol 48: 1611–8. 10.1128/JCM.02243-09 20220175 PMC2863911

[13] Azzari C, Moriondo M, Indolfi G, Cortimiglia M, Canessa C, Becciolini L, et al (2010) Realtime PCR is more sensitive than multiplex PCR for diagnosis and serotyping in children with culture negative pneumococcal invasive disease. PLoS One 5: e9282 10.1371/journal.pone.0009282 20174571 PMC2824814

[14] van den Bergh MR, Spijkerman J, Swinnen KM, François NA, Pascal TG, Borys D, et al (2013) Effects of the 10-valent pneumococcal nontypeable *Haemophilus influenzae* protein D-conjugate vaccine on nasopharyngeal bacterial colonization in young children: a randomized controlled trial. Clin Infect Dis 56: e30–9. 10.1093/cid/cis922 23118268 PMC3540043

[15] Ansaldi F, de Florentiis D, Canepa P, Ceravolo A, Rappazzo E, Iudici R, et al (2013) Carriage of *Streptoccoccus pneumoniae* in healthy adults aged 60 years or over in a population with very high and long-lasting pneumococcal conjugate vaccine coverage in children: Rationale and perspectives for PCV13 implementation. Hum Vaccin Immunother 9: 1–7. 10.4161/hv.23606 23292052 PMC3891719

[16] Heffron R. (1979) Pneumococcus carriers (in) Pneumonia with special reference to pneumococcus lobar pneumonia. Cambridge, MA: Harvard Univeristy Press, pp. 342–78.

[17] Mackenzie G. (1941) The Pneumococcus Carrier. Trans Am Clin Climatol Assoc 57: 88–101. 21407630 PMC2242260

[18] White B. (1938) The carrier state (in) The Biology of Pneumococcus. New York, NY: The Commonwealth Fund, pp. 230–7.

[19] White B. (1938) Isolation of pneumococcus (in) The Biology of Pneumococcus. New York, NY: The Commonwealth Fund, pp. 30–64.

[20] Krone CL, van de Groep K, Trzciński K, Sanders EA, Bogaert D. (2013) Immunosenescence and pneumococcal disease: an imbalance in host–pathogen interactions. Lancet Respir Med 2: 141–53. 10.1016/S2213-2600(13)70165-6 24503269

[21] Wyllie AL, Chu MLJN, Schellens MHB, van Engelsdorp Gastelaars J, Jansen MD, van der Ende A, et al (2014) *Streptococcus pneumoniae* in saliva of Dutch primary school children. PLoS One 9: e102045 10.1371/journal.pone.0102045 25013895 PMC4094488

[22] Regev-Yochay G, Raz M, Dagan R, Porat N, Shainberg B, Pinco E, et al (2004) Nasopharyngeal carriage of *Streptococcus pneumoniae* by adults and children in community and family settings. Clin Infect Dis 38: 632–9. 14986245 10.1086/381547

[23] Ridda I, Macintyre CR, Lindley R, McIntyre PB, Brown M, Oftadeh S, et al (2010) Lack of pneumococcal carriage in the hospitalised elderly. Vaccine 28: 3902–4. 10.1016/j.vaccine.2010.03.073 20398618

[24] Palmu A, Kaijalainen T, Saukkoriipi A, Leinonen M, Kilpi TM. (2012) Nasopharyngeal carriage of *Streptococcus pneumoniae* and pneumococcal urine antigen test in healthy elderly subjects. Scand J Infect Dis 44: 433–8. 10.3109/00365548.2011.652162 22263905

[25] Flamaing J, Peetermans W, Vandeven J, Verhaegen J. (2010) Pneumococcal colonization in older persons in a nonoutbreak setting. J Am Geriatr Soc 58: 396–8. 10.1111/j.1532-5415.2009.02700.x 20370873

[26] McCullers JA. (2014) The co-pathogenesis of influenza viruses with bacteria in the lung. Nat Rev Microbiol 12: 252–62. 10.1038/nrmicro3231 24590244

[27] Mina MJ, Klugman KP. (2014) The role of influenza in the severity and transmission of respiratory bacterial disease. Lancet Respir Med 2: 750–63. 10.1016/S2213-2600(14)70131-6 25131494 PMC4823014

[28] Weinberger DM, Klugman KP, Steiner C, Simonsen L, Viboud C. (2015) Association between Respiratory Syncytial Virus activity and pneumococcal disease in infants: A time series analysis of US hospitalization data. PLoS Med 12: e1001776 10.1371/journal.pmed.1001776 25562317 PMC4285401

[29] Pel J. (1965) Proefonderzoek naar de frequentie en de aetiologie van griepachtige ziekten in de winter 1963–1964. Huisarts Wet 18: 321.

[30] Versalovic J. (2011) Manual of clinical microbiology. 10th ed Washington, DC: ASM Press.

[31] Carvalho MDG, Tondella ML, McCaustland K, Weidlich L, McGee L, Mayer LW, et al (2007) Evaluation and improvement of real-time PCR assays targeting *lytA*, *ply*, and *psaA* genes for detection of pneumococcal DNA. J Clin Microbiol 45: 2460–6. 17537936 10.1128/JCM.02498-06 PMC1951257

[32] Pimenta FC, Roundtree A, Soysal A, Bakir M, du Plessis M, Wolter N, et al (2013) Sequential triplex real-time PCR assay for detecting 21 pneumococcal capsular serotypes that account for a high global disease burden. J Clin Microbiol 51: 647–52. 10.1128/JCM.02927-12 23224094 PMC3553924

[33] Magomani V, Wolter N, Tempia S, du Plessis M, de Gouveia L, von Gottberg A. (2014) Challenges of using molecular serotyping for surveillance of pneumococcal disease. J Clin Microbiol 52: 3271–6. 10.1128/JCM.01061-14 24958802 PMC4313149

[34] Mina MJ, Mccullers JA, Klugman KP. (2014) Live attenuated influenza vaccine enhances colonization of *Streptococcus pneumoniae* and *Staphylococcus aureus* in mice. mBio 5: e01040–13. 10.1128/mBio.01040-13 24549845 PMC3944816

[35] Siegel SJ, Roche AM, Weiser JN. (2014) Influenza promotes pneumococcal growth during coinfection by providing host sialylated substrates as a nutrient source. Cell Host Microbe 16: 55–67. 10.1016/j.chom.2014.06.005 25011108 PMC4096718

[36] McCullers J, Rehg J. (2002) Lethal synergism between influenza virus and *Streptococcus pneumoniae*: characterization of a mouse model and the role of platelet-activating factor receptor. J Infect Dis 186: 341–50. 12134230 10.1086/341462

[37] Wolter N, Tempia S, Cohen C, Madhi S, Venter M, Moyes J, et al (2014) High nasopharyngeal pneumococcal density, increased by viral coinfection, is associated with invasive pneumococcal pneumonia. J Infect Dis 210: 1649–57. 10.1093/infdis/jiu326 24907383

[38] Dhoubhadel BG, Yasunami M, Yoshida L-M, Thi HAN, Thi THV, Thi TAN, et al (2014) A novel high-throughput method for molecular serotyping and serotype-specific quantification of *Streptococcus pneumoniae* using a nanofluidic real-time PCR system. J Med Microbiol 63: 528–39. 10.1099/jmm.0.071464-0 24464695

[39] Webster LT, Hughes TP. (1931) The epidemiology of pneumococcus infection: The incidence and spread of pneumococci in the nasal passages and throats of healthy persons. J Exp Med 53: 535–52. 19869863 10.1084/jem.53.4.535 PMC2131981

[40] Spijkerman J, Prevaes SMPJ, van Gils EJM, Veenhoven RH, Bruin JP, Bogaert D, et al (2012) Long-term effects of pneumococcal conjugate vaccine on nasopharyngeal carriage of *S*. *pneumoniae*, *S*. *aureus*, *H*. *influenzae* and *M*. *catarrhali*s. PLoS One 7: e39730 10.1371/journal.pone.0039730 22761879 PMC3382588

[41] Carvalho MDG, Pimenta FC, Moura I, Roundtree A, Gertz RE, Li Z, et al (2013) Non-pneumococcal mitis-group streptococci confound detection of pneumococcal capsular serotype-specific loci in upper respiratory tract. PeerJ 1: e97 10.7717/peerj.97 23825797 PMC3698467

[42] Carvalho MDG, Bigogo GM, Junghae M, Pimenta FC, Moura I, Roundtree A, et al (2012) Potential nonpneumococcal confounding of PCR-based determination of serotype in carriage. J Clin Microbiol 50: 3146–7. 10.1128/JCM.01505-12 22760044 PMC3421822

