# Supplementary material for: Carriage of Streptococcus pneumoniae in Aged Adults with Influenza-Like-Illness
Source: PLoS One. 2015 Mar 19;10(3):e0119875. doi: 10.1371/journal.pone.0119875 (PMC4366201; doi:10.1371/journal.pone.0119875)
Supplement: S1 Fig — Each dot or cross represents an individual sample. The position of symbols corresponds to CT values for lytA-specific signals in DNA extracted from raw and culture-enriched sample of saliva as marked on corresponding axes. Dots represent 76 saliva samples classified as positive and crosses represent 194 saliva samples classified as negative for S. pneumoniae in the study. Open dots represent 32 saliva samples classified as positive for S. pneumoniae in both raw and culture-enriched samples. Green dots represent 44 samples classified as positive only after culture-enrichment. Dotted lines mark the threshold assigned to discriminate between positive (CT <40) and negative samples, and the total number of 45 cycles in the qPCR reaction. There was a significantly higher number of saliva samples classified as positive for S. pneumoniae after culture-enrichment compared to raw saliva samples (76 or 29% versus 31 or 11%; Fisher’s exact, p<0.001). Culture-enrichment increased the signal strength of the genes targeted by qPCR, with an average overall increase of 2.10 CT for the lytA gene (maximum observed increase of 23.11 CT) and of overall increase of 1.90 CT for the piaA (maximum increase of 21.30 CT). In the subset of 76 saliva samples identified as positive either in raw or culture-enriched sample an average increase for lytA was 6.92 CT and for piaA was 6.64 CT. (PDF) [file pone.0119875.s001.pdf]

Figure S1. Impact of culture-enrichment on *S. pneumoniae* gene *lytA* detection by qPCR in saliva samples (n=270) from elderly.

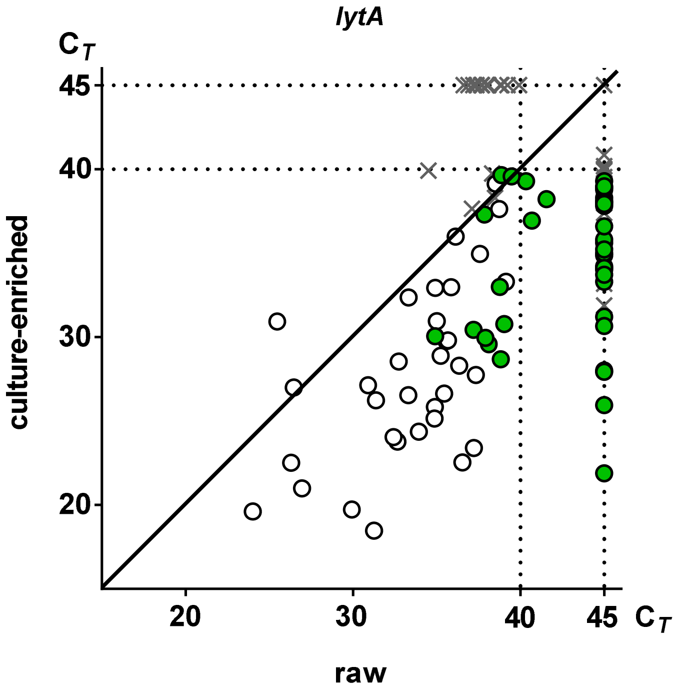

Each dot or cross represents an individual sample. The position of symbols corresponds to C_T values for *lytA*-specific signals in DNA extracted from raw and culture-enriched sample of saliva as marked on corresponding axes. Dots represent 76 saliva samples classified as positive and crosses represent 194 saliva samples classified as negative for *S. pneumoniae* in the study. Open dots represent 32 saliva samples classified as positive *for S. pneumoniae* in both raw and culture-enriched samples. Green dots represent 44 samples classified as positive only after culture-enrichment. Dotted lines mark the threshold assigned to discriminate between positive (C_T<40) and negative samples, and the total number of 45 cycles in qPCR reaction. There was a significantly higher number of saliva samples classified as positive for *S. pneumoniae* after culture-enrichment compared to raw saliva samples (76 or 29% versus 31 or 11%; Fisher's exact, p<0.001). Culture-enrichment increased the signal strength of the genes targeted with qPCR, with an average overall increase of 2.10 C_T for the *lytA* gene (maximum observed increase of 23.11 C_T) and of overall increase of 1.90 C_T for the *piaA* (maximum increase of 21.30 C_T). In the subset of 76 saliva samples identified as positive either in raw or culture-enriched sample an average increase for *lytA* was 6.92 C_T and for *piaA* was 6.64 C_T.
