# Supplementary material for: Carriage of Streptococcus pneumoniae in Aged Adults with Influenza-Like-Illness
Source: PLoS One. 2015 Mar 19;10(3):e0119875. doi: 10.1371/journal.pone.0119875 (PMC4366201; doi:10.1371/journal.pone.0119875)
Supplement: S2 Fig — Each dot represents an individual sample. The position of symbols corresponds to CT values for lytA-specific signals in DNA extracted from raw sample of saliva at ILI onset or after recovery, as marked on X-axis. Horizontal lines represent mean CT value. There was no significant difference between quantities of lytA detected in samples collected at ILI (n = 135) versus the post-recovery sampling time point (n = 135; Mann-Whitney, p = 0.36), neither for the subset of samples considered in the study as positive for S. pneumoniae by qPCR at ILI (n = 42) versus post-recovery (n = 34; p = 0.26). (PDF) [file pone.0119875.s002.pdf]

**Figure S2. Absolute abundance of *Streptococcus pneumoniae* in saliva samples collected at ILI onset and after recovery from ILI.**

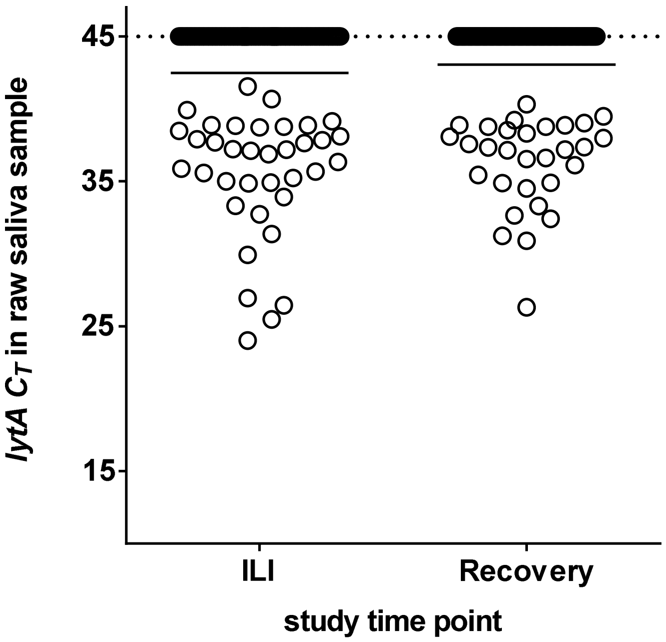


Each dot represents an individual sample. The position of symbols corresponds to $C_T$ values for *lytA*-specific signals in DNA extracted from raw sample of saliva at ILI onset or after recovery, as marked on X-axis. Horizontal lines represent mean $C_T$ value. There was no significant difference between quantities of *lytA* detected in samples collected at ILI (n=135) versus the post-recovery sampling time point (n=135; Mann-Whitney, p=0.36), neither for the subset of samples considered in the study as positive for *S. pneumoniae* by qPCR at ILI (n=42) versus post-recovery (n=34; p=0.26).
